# Chlorpromazine-tadalafil interaction leading to refractory ischemic priapism and penile prosthesis implantation: a case report

**DOI:** 10.1186/s12610-025-00278-x

**Published:** 2025-08-14

**Authors:** Can Arici, Mert Basaranoglu, Selahittin Cayan, Murat Bozlu, Erdem Akbay

**Affiliations:** Department of Urology, School of Medicine, Mersin University, Çiftlikköy Kampüsü, 33343 Yenişehir, Mersin, Turkey

**Keywords:** Priapism, Chlorpromazine, Tadalafil, Drug interaction, Penile prosthesis, Ischemic priapism, Priapisme, Chlorpromazine, Tadalafil, Interaction médicamenteuse, Prothèse pénienne, Priapisme ischémique

## Abstract

**Background:**

Priapism represents a rare urological emergency characterized by persistent penile erection unrelated to sexual stimulation. Although chlorpromazine-induced priapism has been documented, the synergistic interaction with phosphodiesterase type 5 inhibitors resulting in refractory cases necessitating penile prosthesis implantation constitutes a novel clinical scenario requiring comprehensive documentation.

**Case presentation:**

We report a 56-year-old male who developed refractory ischemic priapism following self-administration of oral chlorpromazine for hiccups, taken 24 h after tadalafil 20 mg for erectile dysfunction. The patient had previously experienced a transient priapism episode 10 years earlier following isolated chlorpromazine use, establishing chlorpromazine as the primary etiological agent. Despite comprehensive management including corporal aspiration, intracavernosal sympathomimetic injection, and distal T-shunt creation, the patient developed recurrent priapism requiring penile prosthesis implantation.

**Conclusions:**

This case demonstrates compelling evidence of a dangerous chlorpromazine-tadalafil interaction resulting in treatment-refractory priapism. The synergistic pharmacological effects of alpha-adrenergic blockade and phosphodiesterase type 5 inhibition created a severe clinical presentation necessitating immediate penile prosthesis implantation. Healthcare practitioners must recognize this potentially devastating drug interaction and implement preventive measures through comprehensive medication reconciliation and patient counseling.

## Résumé

**Contexte:**

Le priapisme représente une urgence urologique rare caractérisée par une érection pénienne persistante non liée à une stimulation sexuelle. Bien que le priapisme induit par la chlorpromazine ait été documenté, l’interaction synergique avec les inhibiteurs de la phosphodiestérase de type 5 entraînant des cas réfractaires nécessitant l’implantation d’une prothèse pénienne constitue un nouveau scénario clinique nécessitant une documentation complète.

**Présentation du cas:**

Nous rapportons le cas d’un homme de 56 ans qui a développé un priapisme ischémique réfractaire après auto-administration de chlorpromazine orale pour un hoquet, prise 24 heures après 20 mg de tadalafil pour une dysfonction érectile. Le patient avait déjà présenté un épisode transitoire de priapisme 10 ans plus tôt à la suite d’une utilisation isolée de chlorpromazine, établissant la chlorpromazine comme l’agent étiologique principal. Malgré une prise en charge complète comprenant une aspiration corporale, une injection intracaverneuse de sympathomimétique, et la création d’un shunt en T distal, le patient a développé un priapisme récurrent nécessitant l’implantation d’une prothèse pénienne.

**Conclusions:**

Ce cas montre des preuves convaincantes d’une interaction dangereuse entre chlorpromazine et tadalafil entraînant un priapisme réfractaire au traitement. Les effets pharmacologiques synergiques du blocage alpha-adrénergique et de l’inhibition de la phosphodiestérase de type 5 ont créé un tableau clinique sévère nécessitant l’implantation immédiate d’une prothèse pénienne. Les professionnels de santé doivent reconnaître cette interaction médicamenteuse potentiellement dévastatrice, et mettre en œuvre des mesures préventives par le biais d’un bilan comparatif complet des médicaments et de conseils aux patients.

## Background

Priapism is defined as persistent penile erection exceeding four hours' duration, occurring independently of sexual stimulation and remaining unresolved following ejaculation [1]. This condition constitutes a urological emergency necessitating prompt diagnosis and intervention to prevent long-term sequelae including erectile dysfunction and penile fibrosis [1, 2]. Priapism is classified into three distinct categories: ischemic (low-flow), non-ischemic (high-flow), and stuttering (recurrent) priapism [2]. Ischemic priapism, representing the most prevalent form, results from venous stasis within the corpora cavernosa, subsequently leading to hypoxia, acidosis, and progressive tissue necrosis [3]. Multiple etiological factors have been identified, encompassing hematological disorders, malignancies, trauma, and pharmacological agents [3, 4]. Among medication-induced cases, antipsychotic agents account for approximately 50% of drug-related priapism incidents [5]. The mechanism primarily involves alpha-1 adrenergic receptor antagonism, which disrupts normal detumescence mechanisms [5, 6]. Chlorpromazine, a first-generation antipsychotic of the phenothiazine class, has been associated with priapism since early reports in the 1970s [7]. Despite this established adverse effect, it remains in clinical use for various indications, including hiccup management, potentially leading to unexpected priapism cases, particularly in self-medication scenarios [7, 8]. Although other safer alternatives for hiccup management exist (including baclofen, metoclopramide, and gabapentin), chlorpromazine remains available over the counter in some regions despite its significant side effect profile [8]. The concurrent use of medications affecting erectile function through different mechanisms may substantially increase priapism risk. Phosphodiesterase type 5 (PDE-5) inhibitors, such as tadalafil, are widely used for erectile dysfunction and function by increasing nitric oxide-mediated vasodilation in the corpus cavernosum. When combined with medications having alpha-adrenergic blocking properties like chlorpromazine, a synergistic effect may occur, potentially resulting in prolonged and refractory priapism [8, 9]. Management of ischemic priapism follows a stepwise approach, beginning with corporal aspiration and irrigation, followed by intracavernosal injection of sympathomimetic agents. When these measures fail, surgical interventions such as shunt procedures are indicated [10]. For refractory cases or those presenting after prolonged duration (> 36 h), early penile prosthesis implantation may preserve penile length and address inevitable erectile dysfunction [10, 11]. Here, we present a case of refractory ischemic priapism induced by the interaction between chlorpromazine and tadalafil that ultimately required penile prosthesis implantation after conventional treatments failed. This case provides strong evidence for the causal relationship between this drug interaction and severe priapism, supported by the patient's prior history with chlorpromazine, clear temporal relationship, and systematic exclusion of alternative etiologies.

## Case presentation

### Clinical history and presentation

A 56-year-old male with an unremarkable medical history presented to our emergency department with painful penile erection persisting for 72 h. The patient reported ingesting tadalafil 20 mg (obtained without prescription) approximately 24 h before priapism onset for erectile dysfunction management. Given tadalafil's established pharmacokinetic profile (17.5-h half-life with clinical effects persisting up to 36 h), the medication remained pharmacologically active when he subsequently self-administered oral chlorpromazine 25 mg for persistent hiccups following an upper respiratory tract infection. The chlorpromazine was procured without prescription from a local pharmacy. Upon detailed questioning, the patient disclosed experiencing a similar but transient priapism episode (approximately 4 h' duration) approximately 10 years previously following chlorpromazine administration for hiccups, without concurrent tadalafil or other medication use. At that time, the erection resolved spontaneously, and he did not seek medical attention. The patient had not recognized the connection between the medication and his previous priapism episode until the current presentation. In the current episode, following a second dose of chlorpromazine 25 mg taken 12 h after the first, he developed a persistent erection that failed to resolve, prompting him to seek medical attention after 72 h of symptoms.

### Diagnostic evaluation

Physical examination revealed an erect, rigid, and tender penis, with the glans penis remaining flaccid. Vital signs were stable with blood pressure of 130/80 mmHg, heart rate of 78 beats per minute, respiratory rate of 16 breaths per minute, and temperature of 36.7 °C. Laboratory investigations, including complete blood count, coagulation profile, renal and liver function tests, were within normal limits. Hemoglobin electrophoresis was performed to rule out sickle cell disease or trait, with negative results.

Comprehensive toxicological screening was performed to exclude alternative etiologies for priapism. The screening panel included recreational drugs (cocaine metabolites [benzoylecgonine], cannabis metabolites [11-nor-9-carboxy-THC], amphetamines, and MDMA), serum ethanol concentration, opioids (morphine, codeine, and synthetic opioid metabolites), sedatives (benzodiazepine metabolites and barbiturates), and other substances (phencyclidine [PCP] and tricyclic antidepressants). All toxicological assessments yielded negative results, confirming that recreational substances did not contribute to the development of priapism in this case.

Corporal blood gas analysis revealed a pH of 6.9, a pO2 of 28 mmHg, and a pCO2 of 60 mmHg, confirming the diagnosis of ischemic priapism.

### Treatment approach

Initial management consisted of corporal aspiration using a 16-gauge butterfly needle, yielding dark, deoxygenated blood. This was followed by intracavernosal injection of 2 cc of 1:100,000 epinephrine solution. Despite these measures, detumescence was not achieved. A second attempt with a higher concentration of epinephrine (1:50,000) was also unsuccessful [10]. These first-line interventions were completed within the first two hours of presentation. After first-line treatments failed, the patient underwent a distal T-shunt procedure within four hours of presentation, following American Urological Association guidelines for management of ischemic priapism [10]. The procedure was performed under local anesthesia, and complete detumescence was initially achieved. Postoperatively, the patient was maintained on a urethral catheter, and regular penile "milking" was performed at scheduled intervals to maintain detumescence. Despite these interventions, priapism recurred approximately 6 h after the shunt procedure. The recurrence was likely due to the persistence of both pharmacological agents in the circulation combined with severe corporal smooth muscle damage from the prolonged ischemia. A second attempt at shunt creation with bilateral cavernosoglanular (AlGhorab) shunts was considered but deemed unlikely to succeed given the prolonged duration of priapism and the failure of the initial shunt procedure. Considering the extended duration of the initial priapism (72 h), we proceeded with penile prosthesis implantation to address both the refractory priapism and the anticipated erectile dysfunction [11, 12]. The decision for prosthesis implantation was made within 12 h of the failed shunt procedure.

### Follow-up and outcomes

The patient underwent a three-piece inflatable penile prosthesis implantation under general anesthesia. The procedure successfully achieved detumescence, and the postoperative course was uneventful. The patient was discharged on the second postoperative day without complications. The implantation procedure effectively addressed both the refractory priapism and the anticipated erectile dysfunction.

At 3-month follow-up, the patient reported satisfactory functional outcomes with the penile prosthesis, with no mechanical issues or pain. The patient was able to successfully engage in sexual intercourse, and he expressed satisfaction with the cosmetic and functional results of the implant. This case was reported to our institutional pharmacovigilance committee and subsequently to the national pharmacovigilance database to alert healthcare providers about this potentially dangerous drug interaction.

### Ethical considerations and informed consent

This case report was prepared after obtaining detailed written informed consent from the patient. The informed consent process included explanation of how the clinical information would be used, assurance of anonymity, potential benefits of sharing the case with the medical community, and the patient's right to withdraw consent at any time. The patient was specifically informed about the publication of his medical condition, treatments received, outcomes, and the importance of reporting medication interactions for future patient safety. The study was conducted in accordance with the Declaration of Helsinki. Patient privacy and confidentiality were maintained throughout the process according to institutional protocols for case publications.

## Discussion

### Causality assessment and drug interaction mechanism

This case demonstrates compelling evidence supporting a causal relationship between the combination of chlorpromazine and tadalafil and the development of severe, refractory priapism. Multiple factors substantiate this causality: (1) clear temporal association between drug administration and symptom onset; (2) positive rechallenge with chlorpromazine producing similar symptomatology on two separate occasions separated by 10 years (with the initial episode involving chlorpromazine monotherapy); (3) biological plausibility based on established pharmacological mechanisms of both agents; (4) systematic exclusion of alternative etiologies; and (5) consistency with previously published case reports of similar drug interactions [5, 8, 9].

Utilizing the Naranjo Adverse Drug Reaction Probability Scale, our case achieved a score of 9 points (definite adverse drug reaction): previous conclusive reports (+ 1), adverse event appeared following suspected drug administration (+ 2), adverse reaction improved upon drug discontinuation (+ 1), reaction reappeared upon drug readministration (+ 2), alternative causes systematically excluded (+ 2), reaction with placebo (0), drug detected in toxic concentrations (0, as serum drug levels were not measured), reaction demonstrated increased severity with higher dosage (+ 1), similar reaction to identical drug in patient's history (+ 1), and adverse event confirmed by objective evidence (+ 1) [13].

The pharmacological interaction between chlorpromazine and tadalafil likely created synergistic effects through complementary mechanisms. Chlorpromazine primarily acts as an alpha-1 adrenergic receptor antagonist, inhibiting normal detumescence, while tadalafil increases nitric oxide-mediated vasodilation through PDE-5 inhibition. Additionally, a pharmacokinetic interaction may have occurred, as chlorpromazine can inhibit CYP3A4, which metabolizes tadalafil [6, 9, 14]. Although we could not obtain serum concentrations of chlorpromazine or tadalafil due to the emergency nature of the presentation and lack of immediate laboratory capabilities, the timing of medication use and symptom onset, along with the known pharmacokinetic profiles of both drugs (tadalafil half-life of 17.5 h with effects lasting up to 36 h; chlorpromazine half-life of 30 h), strongly suggest overlapping drug activity that significantly impaired normal erectile physiology regulation. The temporal relationship between drug administration and symptom onset, combined with the established pharmacological interaction mechanisms, strongly supports our hypothesis.

The dose–response relationship was evident, as the patient's initial priapism episode resolved spontaneously after the first dose of chlorpromazine but became persistent after the second dose. This aligns with the pharmacokinetics of both medications, which have relatively long half-lives allowing for drug accumulation with repeated dosing [14, 15].

### Treatment algorithm and decision-making process

The management of this patient followed a systematic, evidence-based approach in accordance with current guidelines. The American Urological Association and European Association of Urology guidelines recommend a stepwise approach to ischemic priapism [10, 16], which was meticulously followed in this case:

1. First-line interventions: Corporal aspiration and irrigation were performed, followed by intracavernosal injection of sympathomimetic agents (epinephrine). These interventions are recommended as initial management with a Grade A recommendation level [10].
2. Second-line interventions: When first-line treatments failed, a distal T-shunt procedure was performed, the recommended surgical intervention for cases refractory to pharmacological management (Grade B recommendation) [10].
3. Decision for penile prosthesis implantation: The decision to proceed with penile prosthesis implantation was based on several factors indicating poor prognosis for spontaneous recovery:
  ◦ Prolonged duration of priapism (72 h), far exceeding the 24–36 h window associated with inevitable erectile dysfunction
  ◦ Failure of both first-line (aspiration and sympathomimetics) and second-line (shunt) interventions
  ◦ Recurrence of priapism after initially successful shunt procedure
  ◦ Evidence from recent systematic reviews and meta-analyses supporting early implantation in refractory cases [11, 17]

The timing of penile prosthesis implantation in this case (after 72 + hours of priapism) is supported by multiple studies. Zacharakis et al. reported that delayed implantation was associated with significantly higher complication rates compared to early implantation [17]. Similarly, Ralph et al. demonstrated that early implantation resulted in better preservation of penile length and lower rates of revision surgery compared to delayed implantation [18].

We considered alternative approaches such as proximal shunts, observation with delayed implantation, or additional pharmacological interventions but rejected them due to the prolonged priapism duration, failure of initial treatments, and low success rates reported in similar cases [10, 16, 18].

### Clinical implications and recommendations

Based on this clinical experience, we recommend several key interventions for healthcare practitioners to prevent and manage similar drug interactions:

Firstly, comprehensive medication histories with particular attention to PDE-5 inhibitor usage should be obtained when evaluating priapism. Patients utilizing chlorpromazine, particularly for non-psychiatric indications such as hiccup management, should receive explicit counseling regarding priapism risk and advised against concurrent PDE-5 inhibitor use. For hiccup management, safer therapeutic alternatives including baclofen (10 mg three times daily) or metoclopramide (10 mg three times daily) should be considered as first-line agents prior to chlorpromazine initiation.

When concurrent administration of these medications becomes necessary, lower initial antipsychotic dosages (e.g., chlorpromazine 10 mg versus 25 mg) and strategic scheduling to minimize pharmacokinetic overlap are advisable. For patients receiving tadalafil therapy, a minimum 48–72 h interval should be recommended before chlorpromazine initiation to minimize interaction risk. For patients presenting with priapism in the context of this drug interaction, clinicians should consider more aggressive early intervention, including expedited surgical management if standard approaches prove ineffective.

Finally, reporting such cases to pharmacovigilance systems facilitates establishment of clearer risk profiles and improves patient safety. We reported this case to our national pharmacovigilance database to contribute to the collective understanding of this drug interaction.

Our case also highlights the risks associated with self-medication, particularly involving drugs with significant adverse effect profiles and interaction potential. Physicians should educate patients about these risks when prescribing medications with known potential for serious adverse effects. Additionally, regulatory authorities should reconsider the over-the-counter availability of medications with significant side effect profiles like chlorpromazine.

## Conclusions

This case demonstrates robust evidence supporting a causal relationship between chlorpromazine as the primary etiological agent and priapism development, with tadalafil co-administration resulting in a more severe, refractory condition necessitating penile prosthesis implantation. The patient's documented history of chlorpromazine-induced priapism without tadalafil co-administration 10 years previously establishes chlorpromazine as the causative agent, while tadalafil likely exacerbated the condition through synergistic pharmacological effects.

This case underscores the critical importance of recognizing potentially catastrophic drug interactions affecting erectile physiology and demonstrates the value of evidence-based guideline adherence in managing refractory priapism. Early consideration of penile prosthesis implantation is warranted in cases of prolonged, treatment-refractory priapism to preserve sexual function and prevent long-term complications.

Healthcare practitioners must maintain heightened vigilance for drug interactions in priapism cases, particularly involving concurrent PDE-5 inhibitor and alpha-blocking agent use. Comprehensive patient education and meticulous medication reconciliation represent essential components of preventing this urological emergency.

### Limitations of the study

This case report possesses several inherent limitations requiring acknowledgment. Firstly, as a single-case study, our findings cannot be extrapolated to broader patient populations, and the strength of evidence remains limited compared to controlled studies or larger case series. Secondly, the lack of serum chlorpromazine and tadalafil concentration measurements represents a significant limitation, preventing direct pharmacokinetic confirmation of the proposed drug interaction mechanism. This absence of quantitative drug level data limits our ability to establish definitive causal relationships and precludes assessment of potential dose-dependent effects. Thirdly, genetic polymorphisms affecting drug metabolism (particularly CYP3A4 variants) were not assessed, which could have influenced individual susceptibility to this drug interaction. Fourthly, the self-medication nature of this case introduces potential inaccuracies in dosing and timing information, relying exclusively on patient recollection. Fifthly, long-term functional outcomes beyond the 3-month follow-up period were unavailable, limiting assessment of prosthesis durability and patient satisfaction over extended periods. Finally, alternative management strategies including different surgical shunt techniques or delayed prosthesis implantation were not explored, potentially limiting the generalizability of our therapeutic approach to similar clinical scenarios.

## Data Availability

No datasets were generated or analysed during the current study.

## Abbreviations

CYP3A4: Cytochrome P450 3A4
PDE-5: Phosphodiesterase type 5
